# Extracellular control of intracellular drug release for enhanced safety of anti-cancer chemotherapy

**DOI:** 10.1038/srep28596

**Published:** 2016-06-23

**Authors:** Qian Zhu, Haixia Qi, Ziyan Long, Shang Liu, Zhen Huang, Junfeng Zhang, Chunming Wang, Lei Dong

**Affiliations:** 1 State Key Laboratory of Pharmaceutical Biotechnology, School of Life Sciences, Nanjing University, 163 Xianlin Avenue, Nanjing 210093, China; 2 State Key Laboratory of Quality Research in Chinese Medicine, Institute of Chinese Medical Sciences, University of Macau, Taipa, Macau SAR

## Abstract

The difficulty of controlling drug release at an intracellular level remains a key challenge for maximising drug safety and efficacy. We demonstrate herein a new, efficient and convenient approach to extracellularly control the intracellular release of doxorubicin (DOX), by designing a delivery system that harnesses the interactions between the system and a particular set of cellular machinery. By simply adding a small-molecule chemical into the cell medium, we could lower the release rate of DOX in the cytosol, and thereby increase its accumulation in the nuclei while decreasing its presence at mitochondria. Delivery of DOX with this system effectively prevented DOX-induced mitochondria damage that is the main mechanism of its toxicity, while exerting the maximum efficacy of this anti-cancer chemotherapeutic agent. The present study sheds light on the design of drug delivery systems for extracellular control of intracellular drug delivery, with immediate therapeutic implications.

The emerging development of drug delivery systems (DDS) has provided various tools for better controlled drug release in the body, with aims to enhance both the efficacy and safety of therapeutic agents^1^. However, most controls have been achieved only at an extracellular level, such as increasing drug accumulation in a certain tissue or improving the drug release profile in the circulation^2^. Few approaches have managed to control the intracellular behaviour of the delivered drug molecules, which are usually ‘burst’ released from the carrier into the cytosol, rapidly accumulating in high concentrations, and randomly interacting with undesignated molecular targets^3^. Such off-target interactions unavoidably bring about side effects and compromise the therapeutic potential of the drugs. Therefore, it is highly desirable to design a delivery system that allows for convenient, extracellular control of intracellular drug release.

Since many therapeutic agents have varying but unique features to interact with multiple cellular components/machineries, which in turn dictate the intracellular fate of these exogenous molecules, successful control of intracellular release of a specific drug may be achieved by intracellular (de-)activation of a specific cellular machinery that ‘ties up’ this drug in the cells. For example, doxorubicin (DOX) is a powerful cancer chemotherapeutic agent with notable nucleic acid-intercalating capability^4^. If we could utilise this feature of DOX and further regulate the cellular machinery that processes nucleic acids, we may achieve the extracellular control of intracellular release of DOX in a reliable manner.

Here, we demonstrate a new approach to meet this challenge, by devising a delivery system to recruit, harness, and then extracellularly manipulate a specific set of cellular machinery that functions as miRNA/siRNA-based gene expression regulation system in eukaryotic cells (Fig. 1). In our design, firstly, DOX intercalates into a double-stranded miRNA mimics, which forms a complex vehicle system HPMD (HSA/PEI/miRNA/DOX), when combined with polyethyleneimine (PEI, 25 kDa, linear) and human serum albumin (HSA). Upon entering the cytosol, the miRNA can recruit a number of proteins including Dicer, R2D2, and Argonaute 2 (Ago2), forming a complex. Secondly, among these proteins, Ago2 triggers the formation of an active RNA-induced silencing complex (RISC) that further recognises and digests target mRNAs, a mechanism we would use in this study to release DOX intracellularly^5^. Thirdly, the activity of Ago2 can be inhibited by the extracellular addition of aurintricarboxylic acid (ATA)^6^, a small-molecule chemical that can curb the intracellular release of DOX. In doing so, we implemented both triggering and inhibitory regulation of Ago2, in order to achieve control over the intracellular release rate of DOX, via simple addition of a small-molecule chemical in the cell medium. In this study, we designed, fabricated, and examined both the safety and efficacy of this delivery system in both *in vitro* cell culture and *in vivo* tumour models.

## Results and Discussion

### Control of the breakdown of miR-16/DOX complex by ATA

**Figure 2:**
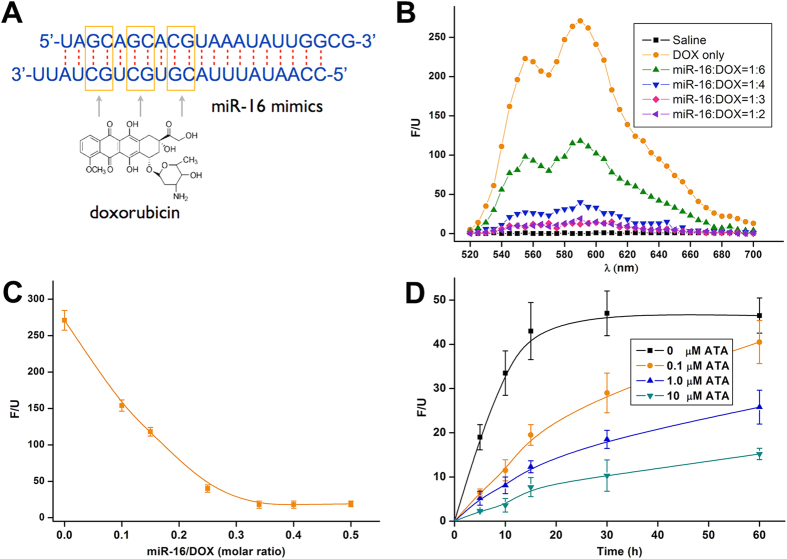
Evaluation of the ATA-responsive miR-16/DOX intercalation. (**A**) Possible sites where DOX inserts into the double strands of the miR-16 mimics. (**B**) Fluorescent spectra of DOX (exciting: 480 nm; emission: 590 nm) in free or intercalated forms. (**C**) Fluorescence absorbance of DOX intercalated with the miR-16 mimics in different molar ratios. (**D**) The release profile of free DOX from the intercalation (miR-16/DOX = 1:3) in response to ATA in different doses; the values are presented as mean of 3 independent experiments ± SEM.

The intercalation between DOX and miRNA constitutes the core of the delivery system. We sought to exploit DOX’s ability to insert into the -GC- base pairs of the RNA double strand in order to form a relatively stable complex. The formed intercalation generally exhibited the property of the RNA molecules^7^, such that we could deliver it into cells by using common RNA-delivering vehicles, such as cationic polymers. Here, we synthesised a segment of nucleic acid mimicking miR-16, a miRNA that is particularly stable under physiological conditions and abundant in serum and cytosol^8^, to intercalate DOX. One miR-16 molecule has 3-GC-pairs in its double strand and can, theoretically, complex up to 3 DOX molecules (Fig. 2A). Free DOX molecules emit fluorescence that can be quenched after intercalation into -GC- sequence^9^, which allowed us to probe the binding between DOX and miRNA with fluorescent spectroscopy. Indeed, we observed decreasing fluorescent intensities of DOX incubated with increasing amounts of miR-16, until the fluorescence was completely quenched when the molar ratio of miR-16 to DOX exceeded 1:3 (Fig. 2B,C), a ratio that correlates well with the theoretical values.

ATA served as the key regulatory mechanism for our delivery system. We then examined whether this small molecule could efficiently inhibit the breakdown of the miR-16/DOX intercalation. We found that in the absence of ATA, DOX was rapidly released from the nucleic acid in cell lysate, with its fluorescent intensity peaked within 20 minutes – a finding consistent with previous reports about the activity of Ago2. However, the addition of ATA into the system effectively curbed such release in a dose-dependent manner; ATA at only 1.0 μM successfully halved the fluorescent intensity (Fig. 2D). These results confirmed that ATA could act as a potent inhibitor, serving to slow down the release of DOX.

To compare the uptake of DOX by mitochondria and nucleus, we purified these two organelles from murine myocardiac cells and incubated them with DOX in different concentrations. At a relatively low concentration (<5 μM), DOX entered nucleus more easily than mitochondria; but, as its concentration increased, DOX quickly accumulated in mitochondria (Supplementary Fig. S1). This result suggests that maintenance of a low DOX concentration in the cytosol could effectively prevent DOX entering mitochondria, which is consistent with a previous study on the mitochondrial toxicity of DOX^10^.

### Control of DOX release from HSA/PEI/miR-16/DOX complex (HPMD) by ATA *in vitro* and *in vivo*

**Figure 3:**
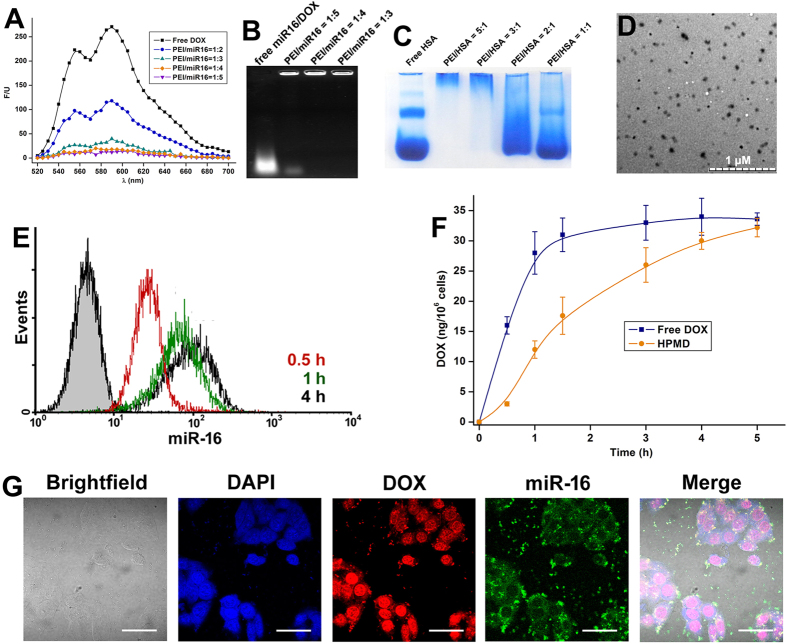
The assembly of HPMD and its transfection *in vitro*. (**A**) The fluorescent spectra of DOX when miR-16/DOX intercalation was combined by PEI in different ratios. (**B**) Electrophoresis of miR-16/DOX intercalation combined by PEI. (**C**) PAGE of HSA complexed with PEI in different ratios. (**D**) TEM observation of HPMD (HSA/PEI/miR-16/DOX = 3:1:4:12). (**E**) FACS examination of fluorescence-labeled miR-16 in HepG2 cells incubated with HPMD (The gray-area curve represents control). (**F**) DOX concentration in HepG2 cells incubated with HPMD or free DOX in 5 hours. (**G**) Confocal image of miR-16 and DOX in HepG2 cells treated with HPMD.

Despite its high stability and binding efficiency, the miR-16/DOX intercalation was unable to enter cells on its own. Based upon our previous studies, we compounded it with PEI and HSA to form the HSA/PEI/miR-16/DOX (HPMD) system^11^,^12^. PEI is a well-established vehicle for the delivery of nucleic acids, which both condenses the intercalation and facilitates cellular uptake^13^. Albumin is a natural transporter in the circulation, and is capable of helping the PEI/miRNA complex avoid phagocytic filtration^14^. Moreover, albumin-recognising receptors on tumour cells can mediate the endocytosis of HPMD and enhance its distribution in tumour cells^14^. We optimised the ratio between the different components to ensure complete combination and prevent leakage of DOX from the system (Fig. 3A–C), and eventually confirmed a molar ratio of 3:1:4:12 (HSA:PEI:miR-16:DOX) in HPMD that was observed as particles of approximately 50 nm in size (Fig. 3D).

**Figure 4:**
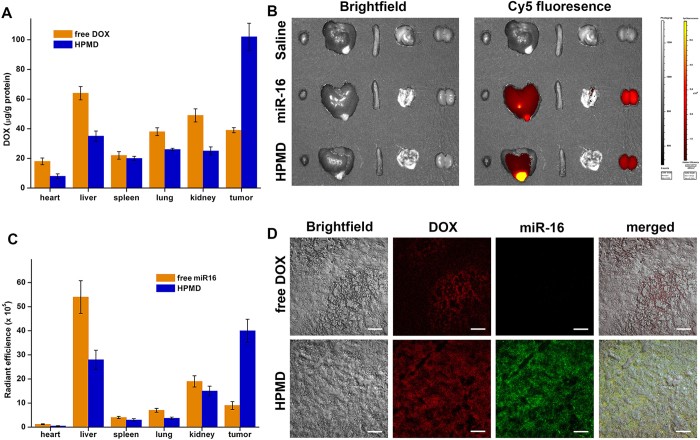
*In vivo* distribution of DOX delivered by HPMD. (**A**) DOX concentration in different organs from mice administered with HPMD or free DOX; n ≥ 7, the values are presented as mean ± SEM. (**B**) Fluorescence of Cy5-labeled miR-16 administered as part of HPMD or free miR-16 and (**C**) the quantified radiant efficiencies imaged and analyzed by an IVIS Lumina XR system; the values are presented as mean ± SEM. (**D**) Confocal analysis of fluorescence-labeled miR-16 and DOX in tumor tissues.

Next, we examined the transfection efficiency of HPMD both *in vitro* and *in vivo*. First, its successful transfection into human hepatoma cell line HepG2 was evidenced by flow cytometry analysis of the intracellular miR-16 pre-labelled with FAM (Fluorescein) (Fig. 3E). Then, we extracted intracellular DOX and quantified its dose by ultraviolet spectroscopy. Both miR-16 and DOX content in the cells reached the maximum after about 4 hours post-transfection; while the intracellular concentration of free DOX peaked within an hour (Fig. 3F). Confocal microscopic examination of the cells incubated with HPMD after 4 hours post transfection revealed that the highest DOX concentration was observed in the nucleus (Fig. 3G). Meanwhile, we saw fluorescent signals for miR-16 in the cytoplasm, which indicated the release of DOX from the miR-16/DOX complex. Additionally, HPMD also exhibited high transfection efficiency *in vivo*. After 12 hours post intravenous administration into an orthotopic, allograft hepatoma model in the mouse liver^15^, the complex significantly enhanced the accumulation of DOX in the tumour and lowered its distribution in other organs, as evidenced by multiple assays – including the measurement of the DOX doses in the tumour and different organs (Fig. 4A), imaging of a Cy5-labelled miR-16 mimics (Fig. 4B), quantification of Cy5 radiation (Fig. 4C), as well as microscopic evaluation of the liver and tumour tissue sections (Fig. 4D). Taken together, HPMD efficiently delivered the miR-16/DOX intercalation into tumour cells both *in vitro* and *in vivo*.

We next tested the ability of ATA to enter the three kinds of cells used in this study: HepG2 cells, Heps cells and mouse primary myocadiac cells, which is the basis for this molecule’s intracellular function. As shown in Supplementary Fig. S2, ATA could be efficiently taken up by the cells. Meanwhile, although there were reports that ATA could suppress cell apoptosis^16^, we found that ATA used in our study (1 μM) had no significant influence on DOX-caused cell death (Supplementary Fig. S3).

**Figure 5:**
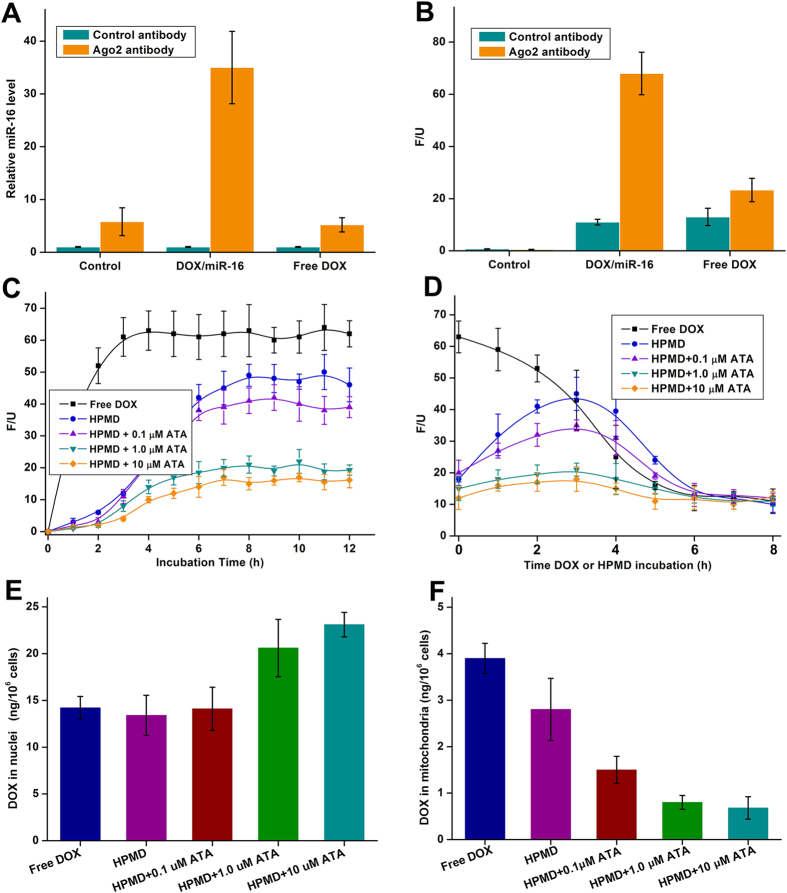
Test of ATA/HPMD in HepG2 cells. (**A,B**) Measurement of the levels of (**A**) miR-16 and (**B**) DOX bound to RISC which was pulled down with an Ago2 antibody from cells treated with free DOX or HPMD. (**C,D**) Fluorescent intensity of DOX in (**C**) cells treated with ATA/HPMD or free DOX for 12 hours and (**D**) cells treated with ATA/HPMD or free DOX for 4 hours and then DOX-free ATA media for another 4 hours. (**E,F**) The DOX concentrations in (**E**) the nuclei and (**F**) mitochondria of cells treated with ATA/HPMD or free DOX for 12 hours. The values are presented as mean of 3 independent experiments ± SEM.

In contrast to delivering free DOX, our strategy to harness the cellular machinery RISC provides a two-fold mechanism to control the release of DOX inside the cells: i) HPMD can integrate into RISC, and Ago2 of RISC triggers the intracellular release of DOX; ii) the activity of Ago2 can further be suppressed by extracellular addition of ATA – a small-molecule inhibitor of Ago2. To examine mechanism i), we transfected a group of cells with HPMD and treated another with free DOX, pulled down RISC with an antibody to Ago2 and performed a co-immunoprecipitation assay. We found that the amounts of both miR-16 (Fig. 5A) and DOX (Fig. 5B) were distinctly higher in the HPMD-transfected cells than in the free DOX-treated group, suggesting that miR-16/DOX bound to RISC in the cells. As discussed above, DOX was efficiently released from HPMD and was able to reach the same maximum intracellular amount of that of free DOX (Fig. 3F). To test mechanism ii), we added ATA (0.1, 1.0, and 10 μM) to the HPMD-transfected cells and determined the level of free DOX by measuring its fluorescence in the cell lysates. As expected, ATA inhibited the release of free DOX in a clear dose-dependent manner (Fig. 5C). To more precisely demonstrate the control of ATA over the release of DOX from HPMD, we cultured the cells with either free DOX or HPMD for 4 hours, removed the media, refilled the wells with DOX-free ATA media, and then measured the fluorescence of DOX from the cell lysate. This enabled us to accurately monitor the action of RISC and ATA without interference with the cells’ continual intake of DOX. As shown in Fig. 5D, in the cells treated with free DOX, the intracellular DOX concentration steadily decreased following the removal of the DOX supply, which could be attributed to the increasing intercalation of DOX molecules into nucleic acids; meanwhile, in the HPMD-transfected cells, the level of free DOX increased in the first 3 hours and then decreased, a trend that reflects a dynamic process comprising the release of DOX from the intercalation and then suppression of the release by the action of ATA. Notably, the release curve was almost completely flattened at 1.0 μM ATA. This suggests that at this concentration, the slow release rate of DOX from RISC allowed for immediate capture of the escaped DOX by nearby double-strand nucleic acids – most probably DNA in the nucleus. To confirm this, we further isolated the nucleus and mitochondria, and quantified the level of DOX in these two organelles. We found that, in the HPMD-transfected cells, the addition of ATA promoted the accumulation of DOX in the nucleus (ATA at 1.0 and 10 μM, Fig. 5E) while prevented its presence in the mitochondria (ATA at all concentrations, Fig. 5F), which is in agreement with previous report that DOX, at a relatively low cytosol dose, preferentially binds DNA in the nucleus rather than entering the enclosed mitochondria^10^.

### Protective effects of ATA/HPMD against reactive oxygen species (ROS) *in vitro*

**Figure 6:**
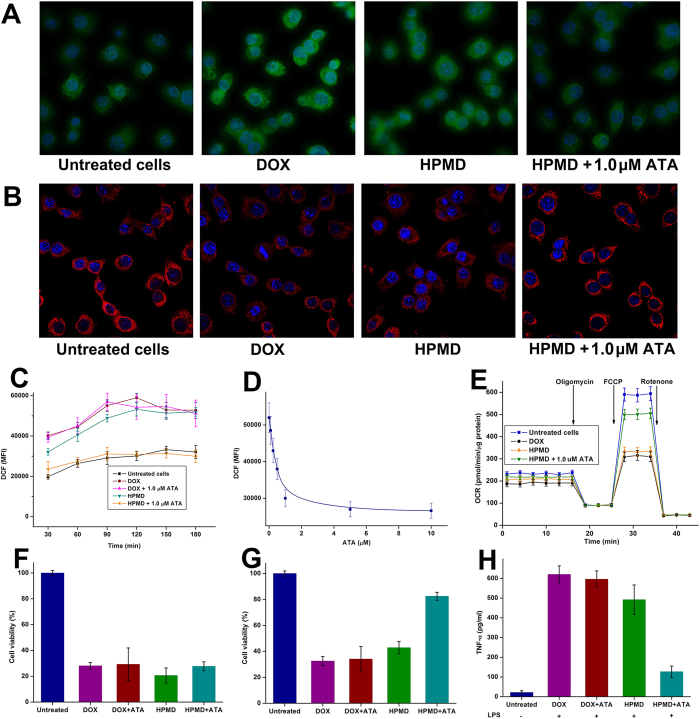
Assessment of the protective effects of ATA/HPMD. (**A,B**) Fluorescent staining for (**A**) reactive oxygen species (ROS) and (**B**) mitochondria of HepG2 cells treated with ATA/HPMD or free DOX for 2 hours. (**C**) Mean fluorescent intensity (MFI) representing the levels of ROS in the cells in 3 hours. (**D**) The relationship between intracellular ROS level and the ATA concentration. (**E**) Oxygen consumption rate (OCR) test of the cells by a Seahorse Analyzer. (**F–G**) Cell viability of (**F**) HepG2 cells and (**G**) primary myocardial cells treated with ATA/HPMD or free DOX for 24 hours. (**H**) The level of TNF-α produced in primary macrophages treated with ATA/HPMD or free DOX for 3 hours and stimulated with LPS for 6 hours.The values in (**C–G**) are presented as mean of 3 independent experiments ± SEM.

Successful control of intracellular drug release may provide timely solutions to overcoming key drawbacks with the delivered drugs. A major concern with the therapeutic use of DOX is that it induces ROS production in mitochondria, which damages its membrane and compromises its functions^17^,^18^. By using the 2′,7′-Dichlorodihydrofluorescein diacetate (DCFH-DA) dye that specifically probes cellular ROS, we demonstrated that the ATA/HPMD system generated significantly lower levels of ROS than free DOX (Fig. 6A). Further measurement of the mean fluorescence intensity (MFI) at different time intervals confirmed that HPMD, under the regulation of ATA, minimised the production of ROS to a level comparable to that of the untreated cells (Fig. 6C); and such an effect of ATA was dose dependent (Fig. 6D). As a result, controlled release of DOX with HPMD effectively prevented damage to mitochondrial membrane, as evidenced by TMRE – a fluorescent probe for mitochondrial membrane (Fig. 6B), and maintained the mitochondrial function, as characterised by the measurement of the oxygen consumption rate (OCR) with a Seahorse Metabolic Analyser (Fig. 6E).

DOX causes cytotoxicity via two major mechanisms – i) intercalating cellular DNA and ii) increasing ROS concentration. As DNA replicates much faster in tumour than in normal cells, it is desirable that the delivered DOX mainly targets DNA but generates minimal level of ROS, so that it can selectively kill tumour cells^19^. HPMD demonstrated this feature. Under the regulation of ATA, HPMD maintained the toxicity of DOX on HepG2 cancer cells (Fig. 6F) but exhibited significantly lower toxicity than free DOX on primary myocardial cells (Fig. 6G). To further validate the effect of ATA/HPMD on the inhibition of ROS release, we challenged primary peritoneal macrophages with lipopolysaccharides (LPS), and found that ATA/HPMD could remarkably decrease the expression of inflammatory cytokine tumor necrosis factor-α (TNF-α) – a typical response of macrophages augmented by DOX-induced ROS (Fig. 6H). These findings demonstrate that HPMD can be both an efficient and – under the extracellular control of ATA – a safer system for the intracellular delivery of DOX.

### *In vivo* toxicity and anti-tumour performance of ATA/HPMD

**Figure 7:**
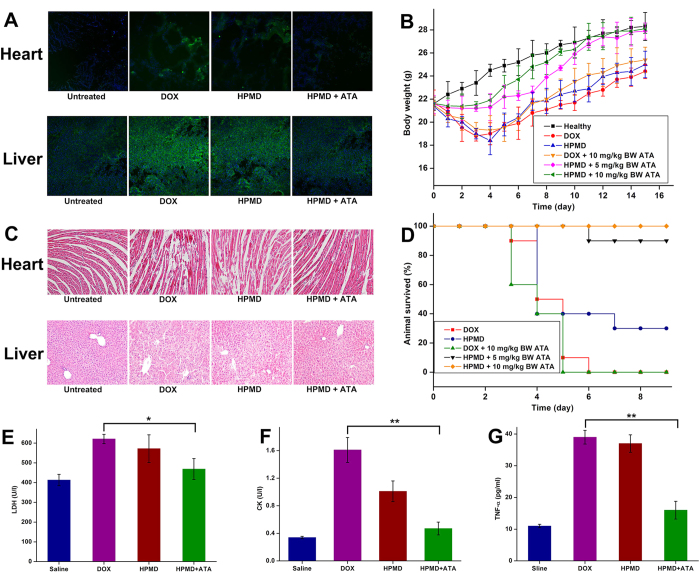
Test of ATA/HPMD *in vivo*. Mice were treated with ATA/HPMD or free DOX (Non-lethal dose: 10 mg/kg body weight, lethal dose: 20 mg/kg body weight) for up to 15 days, followed by (**A**) staining for ROS in heart and liver tissues, (**B**) measurement of body weight, (**C**) histological H&E analysis of heart and liver, (**D**) survival counts, and determination of the serum levels of (**E**) LDH, (**F**) CK, and (**G**) TNF-α. The values in (**B,E–G**) are presented as mean ± SEM, n ≥ 7. *p < 0.05, **p < 0.01.

**Figure 8:**
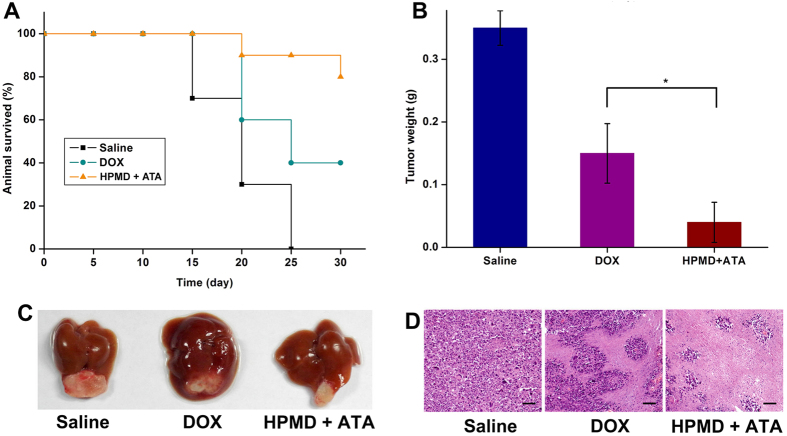
Therapeutic effects of ATA/HPMD in an *in vivo* cancer model. (**A**) Survival rate, (**B**) tumour weights, (**C**) gross view of tumours, and (**D**) H&E histological analysis of the tumours of animals treated with ATA/HPMD or free DOX for 2 weeks.The values in (**B**) are presented as mean ± SEM, n ≥ 7. *p < 0.01.

Delivery with the ATA/HPMD system increased both the safety and efficacy of DOX *in vivo*. In comparison with the delivery of free DOX or uncontrolled HPMD, the ATA-regulated HPMD system dramatically decreased the production of ROS in cardiac and liver tissues (Fig. 7A) and demonstrated protective effects against free DOX-induced damages. This can be evidenced by body weight measurements (Fig. 7B), histological observation of heart and liver tissue (Fig. 7C), animal death counts (Fig. 7D), as well as determination of several tissue damage and inflammatory markers (LDH, Fig. 7E; CK, Fig. 7F; TNF-α, Fig. 7G). We next evaluated the therapeutic efficacy of this system in the mouse liver model. As compared with the delivery of free DOX, the ATA-regulated HPMD system significantly increased the animal survival rate (Fig. 8A) and decreased the tumour weights (Fig. 8B), supported by the images for the gross view of tumour samples (Fig. 8C) as well as histological analysis (Fig. 8D). We speculated that both the accumulation of DOX in tumour sites, as discussed above with data shown in Fig. 4, and the reduced toxicity of DOX contributed to this improvement in terms of therapeutic efficacy.

In conclusion, we have developed a drug delivery system that has met the challenge of controlling the intracellular release of DOX whilst also demonstrating two unique physiological advantages. First, this is a ‘biological’ control that harnesses an inherent biological feature of the delivered drug DOX (i.e. to intercalate DNA/RNA double strand) and an inherent biological function of the cells (i.e. the RISC machinery). In comparison with manipulating the drug carrier to release the drug in response to the changes in enzyme, pH, or temperature^20^,^21^,^22^, our control is direct, convenient (by simply adding small molecules), and less susceptible to any changes in cell microenvironments. Moreover, our design substantially reduced the ROS-relevant toxicity of DOX and thus remarkably increased its safety, a major challenge that has long hindered the extended clinical use of DOX as an anti-cancer chemotherapeutic drug. Meanwhile, under optimal control, HPMD released DOX at a very low rate inside the cells, which facilitated its nucleic enrichment and thereby enhanced its activity to intercalate DNA – as its main mechanism of action. The encouraging performance of this system further highlights that control of intracellular drug release may hold a vital position in future’s design and improvement of drug delivery systems for clinical applications.

## Materials and Methods

### Reagents

Doxorubicin hydrochloride was purchased from Huafeng United Technology Co., Ltd. (Beijing, China). MicroRNA-16 (miR-16) mimics and cyanine 5 (Cy5)-labeled miR-16 mimics were synthesized by Takara Biotechnology Co., Ltd. (Shiga, Japan). Human serum albumin (HSA) was purchased from Oddfoni Biological Technology Co., Ltd. (Nanjing, China). Aurintricarboxylic acid (ATA) was purchased from Aladdin Reagent Co., Ltd. (Shanghai, China). Enzymatic reagent kits for the determination of serum creatine kinase (CK) and lactate dehydrogenase (LDH) were purchased from Jiancheng Bioengineering Institute (Nanjing, China). Polyetherimide (PEI, 25 KDa, linear) and the other reagents used in this study were purchased from Sigma-Aldrich (St. Louis, MO, USA).

### Cells and Animals

Human hepatoma cell line (HepG2) and mouse hepatoma solidity cells (Heps) were obtained from Institute of Biochemistry and Cell Biology, SIBS, CAS (Shanghai, China) and cultured in RPMI-1640 medium, supplemented with 10% FBS (Life Technologies, Grand Island, New York, USA). ICR mice (20 ± 2 g) were purchased from the Experiment Animal Center of Nanjing Medical University (Nanjing, China). All animal experiments in this study were evaluated and approved by the Animal Ethics Review Committee of Nanjing University. The design, practice and termination of these experiments, as well as the animal care throughout, strictly adhered to the Guidelines for the Care and Use of Laboratory Animals of Nanjing University.

Primary mouse myocardial cells was harvested according to a published protocol published^23^. Briefly, the mice were injected with heparin, anesthetized, and perfused with calcium-free perfusion buffer to flush blood from the vasculature. Then, the hearts were perfused with a collagenase solution to digest the extracellular matrix. The digestion upon completion was stopped with serum, and hearts were dissociated gently until all the large pieces of hearts were dispersed in the cell suspension. After centrifugation, the pelleted cells were resuspended and plated on laminin-coated dishes and incubated for 1 h to allow myocyte attachment. These myocytes were cultured in Modified Eagle’s Medium (MEM) with Hanks’ Balanced Salt Solution, supplemented with 1 mg/ml bovine serum albumin and 100 U/ml penicillin at 37 °C in a 2% CO_2_ incubator. Primary mouse peritoneal macrophages were isolated according to a published protocol^24^. Briefly, 10 ml cold 1 × PBS was intraperitoneally injected into euthanized mice, and about 8 ml fluid was aspirated from peritoneum. The peritoneal exudate cells were centrifuged at 400 g for 10 min, 4 °C. Cell pellet was resuspended in cold DMEM and cultured in DMEM with 10% FBS.

The orthotopic hepatic Heps tumor model was created by intrahepatic injection of Heps cell (1 × 10^5^ cells) into anesthetized mice with a microinjector. The mice were kept in a warm cage before recovery and penicillin was given via drinking water for 3 days.

### Preparation and characterization of HPMD

miR-16/DOX intercalation was formed by mixing different amounts of miR-16 with 2 mg/ml DOX to find the DOX:miR-16 ratio at which free DOX can insert into the miR-16 duplex completely. Because the fluorescence of DOX (exciting: 480 nm; emission: 520–700 nm) would quench when it inserts into miR-16, we can determine the formation of the miR-16/DOX complex by measuring the fluorescence of DOX using automatic multifunctional microplate reader (Tacon, Switzerland). PEI was added to miR-16/DOX at different PEI/miR-16 weight ratios to combine the intercalations. The PEI/miR-16/DOX complex was examined for DOX fluorescence detecting and agarose gel electrophoresis (0.8% agarose in TAE buffer). HPMD was obtained by mixing PEI/miR-16/DOX complex solution with the same volume of 2 mg/ml HSA solution by gentle agitation for 1 h. To validate the combination, HPMD with different HSA/PEI weight ratios were subjected to native polyacrylamide gel electrophoresis (PAGE) (without SDS and β-mercaptoethanol)^25^. The albumin in gel was stained with Coomassie brilliant blue R250. The diameters and zeta potential of HPMD were analyzed by dynamic light scattering (DLS) measurements using a 90 Plus Particle Size Analyzer (Brookhaven Instruments, Holtsville, NY). The morphology of the complexes was examined with a transmission electron microscope (TEM, JEOL, Japan).

### *In vitro* DOX release properties of miR-16/DOX intercalation

miR-16/DOX (miR-16/DOX = 1:3) intercalation was digested in a cell lysate extracted from HepG2 according a published method^26^. Different amount of ATA was added into the lysate to inhibit the activity of Ago2. Then the resulting solution was examined by DOX fluorescence.

### Cellular uptake and distribution of HPMD

HepG2 cells (4 × 10^4^ cells) were plated on Lab-Tek II eight-well chambered coverglasses (Nalge Nunc International, Rochester, NY, USA) and 24 h later, cells were transfected with HPMD to a concentration of 0.2 μg/ml DOX in the cell culture. For confocal visualization, the Cyanine 5-labeled miR-16 (Cy5-miR-16) and was used to form HPMD, and the cells were fixed and examined under a laser scanning confocal microscope (Nikon, Japan). The fluorescent signals of DOX (exciting: 480 nm; emission: 590 nm), Cy5-miR-16 (exciting: 650 nm, emission: 670 nm) were discriminated based on their differences in the exciting wavelength by the confocal. In our experiments, the fluorescence of DOX was shown in red and Cy5-miR-16 was shown in green. For FACS examination, the cells were harvested at the indicated times, resuspended in 300 μl 1 × PBS, and analyzed on FACS Calibur (BD Biosciences, San Jose, CA) using Cell Quest software. The results were presented as overlaid histograms. All experiments were triplicated.

### Cellular free DOX measurement

Cells containing DOX were suspended in an appropriate volume of complete immunoprecipitation lysis buffer (20 mM Tris-HCl, pH 7.5, 150 mM NaCl, 0.5% NP-40, 2 mM EDTA, 0.5 mM dithiothreitol (DTT), 1 mM NaF, 1 × protease inhibitor and 1 × PMSF) for 30 min on ice. The free DOX in the lysates was determined for its fluorescence intensity (exciting: 480 nm; emission: 590 nm).

### Cell viability assay

Cell Counting Kit 8 (CCK-8, Dojindo Molecular Technologies, Gaithersburg, MD) assay was performed for determining the effect of ATA/HPMD or free DOX on HepG2 cells and primary myocardial cells growth *in vitro*. Cells were cultured in 96-well plates (1 × 10^4^ cells) and treated with DOX, ATA + DOX, HPMD or ATA + HPMD at the indicated drug dosage. 24 h later, 10 μl CCK-8 was added to each well and cells were incubated at 37 °C for 1–2 h. The experiment was performed in quintuplicate. Absorbance was read at 450 nm with an automatic multifunctional microplate reader.

### Immunoprecipitation assay

The integration of miR-16/DOX intercalation into RISC was verified by immunoprecipitation assay. Cells were resuspended in an appropriate volume of complete immunoprecipitation lysis buffer (20 mM Tris-HCl, pH 7.5, 150 mM NaCl, 0.5% NP-40, 2 mM EDTA, 0.5 mM dithiothreitol (DTT), 1 mM NaF, 1 × protease inhibitor and 1 × PMSF) for 30 min on ice. The lysates were immunoprecipitated with mouse monoclonal anti-AGO2 antibody or mouse normal IgG followed by protein G-Agarose beads. After purification, the immunoprecipitated RNA was extracted with miRNeasy Mini Kit (Qiagen) and analyzed by RT-qPCR using TaqMan miRNA probes (Applied Biosystems), and the DOX in the protein complex was extracted by the chloroform/n-butanol assay and quantified by its absorbance at 240 nm^27^.

### Quantification of DOX in mitochondria and nucleus

The separation and purification of mitochondria and nucleus of the cells were by density gradient centrifugation according a published protocol^28^. Briefly, the collected cells were homogenized using a glass–Teflon potter at 1600 rpm. Then the cell lysate was layered onto a sucrose gradient containing 10 ml 1.5 M sucrose above 20 ml 2.2 M sucrose in 1.5% citric acid, and centrifuged at 20,000 rpm for 30 min. The upper layer (0.25–1.5 M interface) consisted largely of mitochondria, while the lower layer (1.5–2.2 M interface) and the pellet contained nuclei. The DOX were extracted by the chloroform/n-butanol assay^26^ and quantified by its absorbance at 240 nm.

### ROS detection

According to a verified protocol^29^, intracellular ROS levels were measured using DCFA-DA oxidation. For flow cytometric analysis, cells were incubated with 10 μM CM-H2DCFDA (Invitrogen, San Diego, CA) for 10 min. The cells were then analyzed using a FACS Calibur flow cytometer (BD Biosciences, San Jose, CA) using the FL-1 channel (515–545 nm). The mean fluorescence intensity (MFI) was analyzed using CellQuest Pro software, and quantification was performed using WinMDI 2.8 software (The Scripps Institute, La Jolla, CA).

### Mitochondria staining by TMRE staining

According to the method^30^, A TMRE (Invitrogen, Shanghai, China) stock was prepared at a concentration of 20 mg/ml in DMSO and stored at −70 °C. Working stocks of 1.0 mg/ml were made up fresh in distilled water. For estimation of mitochondria, cells were incubated with 100 nM TMRE for 20 min in HEPES-buffered saline (HBS) consisting of 144 mM NaCl, 10 mM HEPES, 2 mM CaCl_2_, 1 mM MgCl_2_, 5 mM KCl and 10 mM D-glucose. TMRE fluorescence was then visualized using a fluorescence microscope (Nikon TE2000; Nikon, Tokyo, Japan). Optics were as follows: excitation, 490 nm and emission, 510 nm.

### Mitochodrial OCR

The mitochondrial function was evaluated by a Seahorse analyzer. Basal OCR was measured over time followed by observing the effects of the mitochondrial inhibitors, oligomycin (1 mg/mL), FCCP (2 mM), and antimycin A (10 mM). ATP linked respiration was derived from the difference between OCR at baseline and respiration following oligomycin addition. The difference in OCR between antimycin A and oligomycin represented the amount of oxygen consumed that is due to proton leak. Maximal OCR was determined by subtracting the OCR after antimycin A addition from the OCR induced by FCCP. Lastly, the reserve capacity was calculated by the difference between maximal (FCCP) and basal respiration.

### Cytokine quantification

TNF-α in the cell culture medium and mouse serum was quantified by an ELISA kit (Abcam, MA, USA.).

### Tissue distribution studies in tumor-bearing mice

Orthotopic Heps-bearing mice were used to study tissue distribution. Free DOX and HPMD were given *via* tail vein at a dose of 20 mg DOX/kg body weight. Different organs were harvested 6 h after injection. DOX in these tissues was extracted and quantified by examining its fluorescence intensity at 590 nm. Cy5-miR-16 was used to form HPMD to facilitate the *in vivo* drug distribution tracing. Then naked DOX, HSA/PEI/Cy5-miR-16, and HPMD were separately injected into tumor-bearing mice *via* tail vein at a dose of 1.5 mg Cy5-miR-16/kg body weight. Six hours after administration, different organs were harvested and imaged by IVIS Lumina XR system (XENOGEN, Caliper, MA, USA). Fluorescence quantification of images was analyzed using IVIS Living Imaging Software. Then, tumors were separated, embedded in OCT, and examined by a confocal microscope.

### Toxicology study

ATA/HPMD and free DOX were intravenously injected into healthy mice at the doses of 10 mg or 20 mg DOX/kg body weight and 5 mg or 10 mg ATA/kg body weight. Mice were given enough pellet food and water. The body weight changes were recorded every day for two weeks. The survival ratio of the animals was also calculated. For histology, different tissues harvested on the 4th day after DOX and HPMD administration were fixed in 4% paraformaldehyde, embedded in paraffin, sectioned, stained with hematoxylin and eosin and analyzed by microscope (Nikon TE2000-U, Japan). The heart and liver tissues were also embedded in OCT for frozen sections, stained with DCFH-DA dye and examined under confocal microscope. The blood samples were centrifuged at 4000 rpm for 10 min at room temperature to collect serum, and then the serum CK and LDH were measured for evaluation of the function of hearts.

### Anti-cancer efficacy evaluation

The antitumor activity of HPMD and free DOX was examined in the orthotopic Heps-bearing mice. One week after injection of Heps cells, mice were randomly divided into three groups and injected intravenously with saline, free DOX or HPMD + ATA at 5 mg DOX/kg body weight and 10 mg ATA/kg body weight every three days for three times. After the treatment, all tumors were separated, weighed and fixed for histological analysis. Having been fixed with 4% paraformaldehyde for 24 h, tissues were embedded in paraffin, sectioned at 5 μm, stained with hematoxylin and eosin and analyzed by microscope (Nikon TE2000-U, Japan). The survival ratio of the animals was also calculated for 30 days.

### Statistical analysis

Data are presented as the mean ± standard error. Statistical analyses were performed using the Student’s t-test for pairs of groups, and one-way ANOVA analysis of variance for multiple groups, with significance set at a p-value < 0.05.

## Additional Information

**How to cite this article**: Zhu, Q. *et al*. Extracellular control of intracellular drug release for enhanced safety of anti-cancer chemotherapy. *Sci. Rep.* **6**, 28596; doi: 10.1038/srep28596 (2016).

## Supplementary Material

Supplementary Information

## Figures and Tables

**Figure 1:**
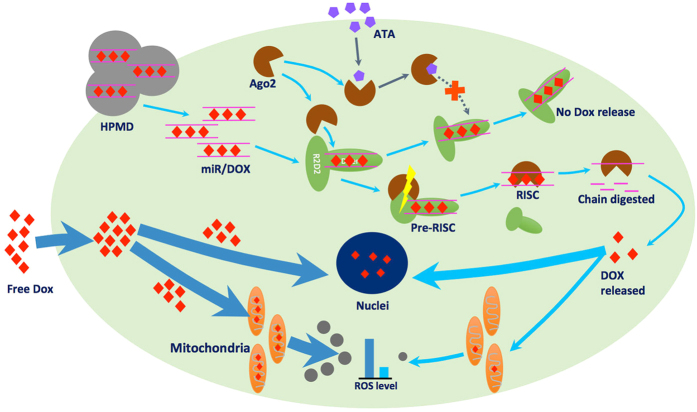
Scheme of the intracellular controlled release of DOX by an extracelluar ATA administration.
