# Supplementary material for: Extracellular control of intracellular drug release for enhanced safety of anti-cancer chemotherapy
Source: Sci Rep. 2016 Jun 23;6:28596. doi: 10.1038/srep28596 (PMC4917837; doi:10.1038/srep28596)
Supplement: Supplementary Information [file srep28596-s1.pdf]

Extracellular control of intracellular drug release for enhanced safety of anti-cancer chemotherapy

Qian Zhu[a], Haixia Qi[a], Ziyan Long[a], Shang Liu[a], Zhen Huang[a], Junfeng Zhang [a], Chunming Wang [b], Lei Dong [a]

[a]State Key Laboratory of Pharmaceutical Biotechnology, School of Life Sciences, Nanjing University, 163 Xianlin Avenue, Nanjing 210093, China.

[b]State Key Laboratory of Quality Research in Chinese Medicine, Institute of Chinese Medical Sciences, University of Macau, Taipa, Macau SAR.

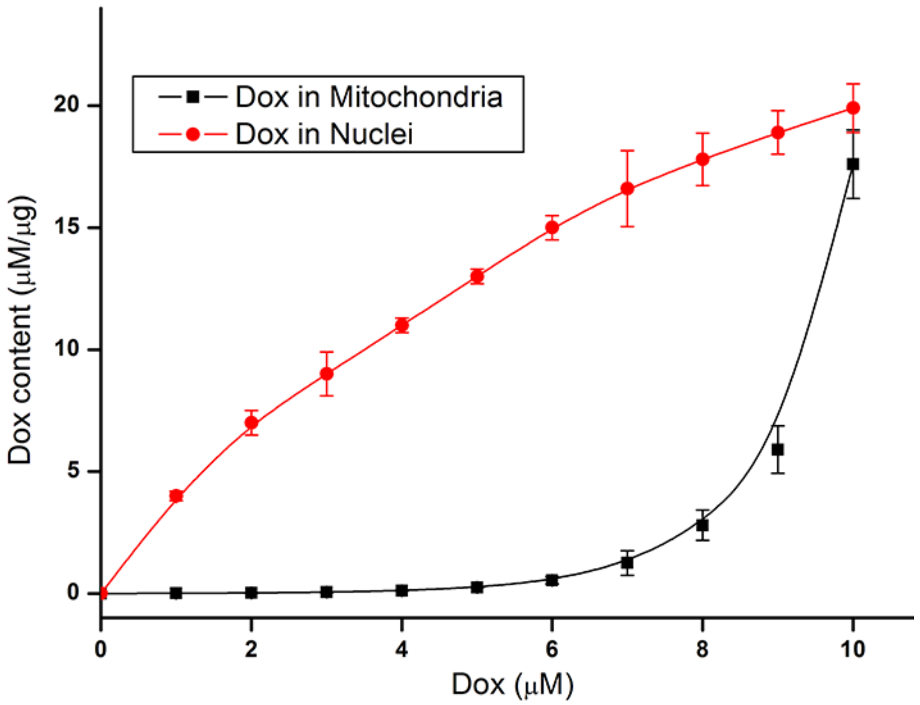

Figure S1: Mitochondria and nucleus were separated from mouse primary myocardial cells and were incubated with Dox of different concentration solved in PBS at 37 ^{o}C for 30 min. Dox contents in mitochondria and nucleus were then quantified. The experiments were triplicated and the results were mean ± SE.

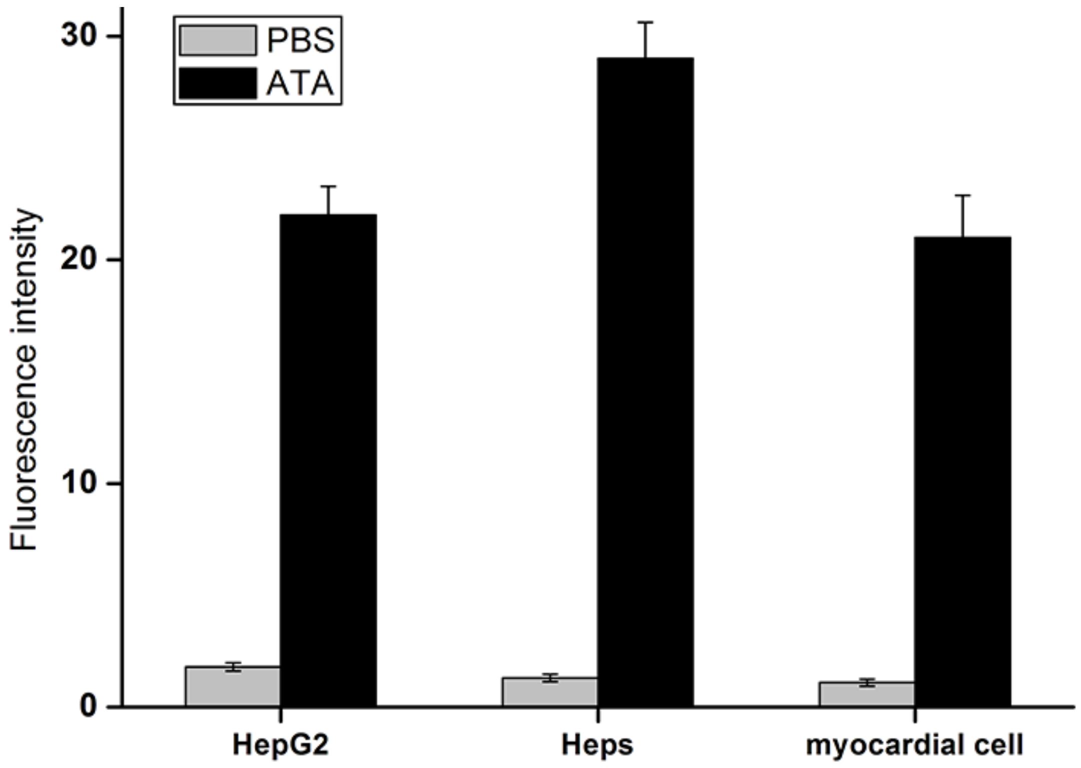

Figure S2: HepG2, Heps and mouse primary myocardial cells (1×10^6) were incubated with 1 μM ATA in PBS at 37 ^{o}C for 30 min. After removal of extracellular ATA, cell lysates were obtained and centrifuged. The supernatants were diluted into 100 μL PBS for examination of ATA fluorescence intensity (Exc: 220 nm, Emi: 409 nm). The experiments were triplicated and the results are expressed as mean ± SE.

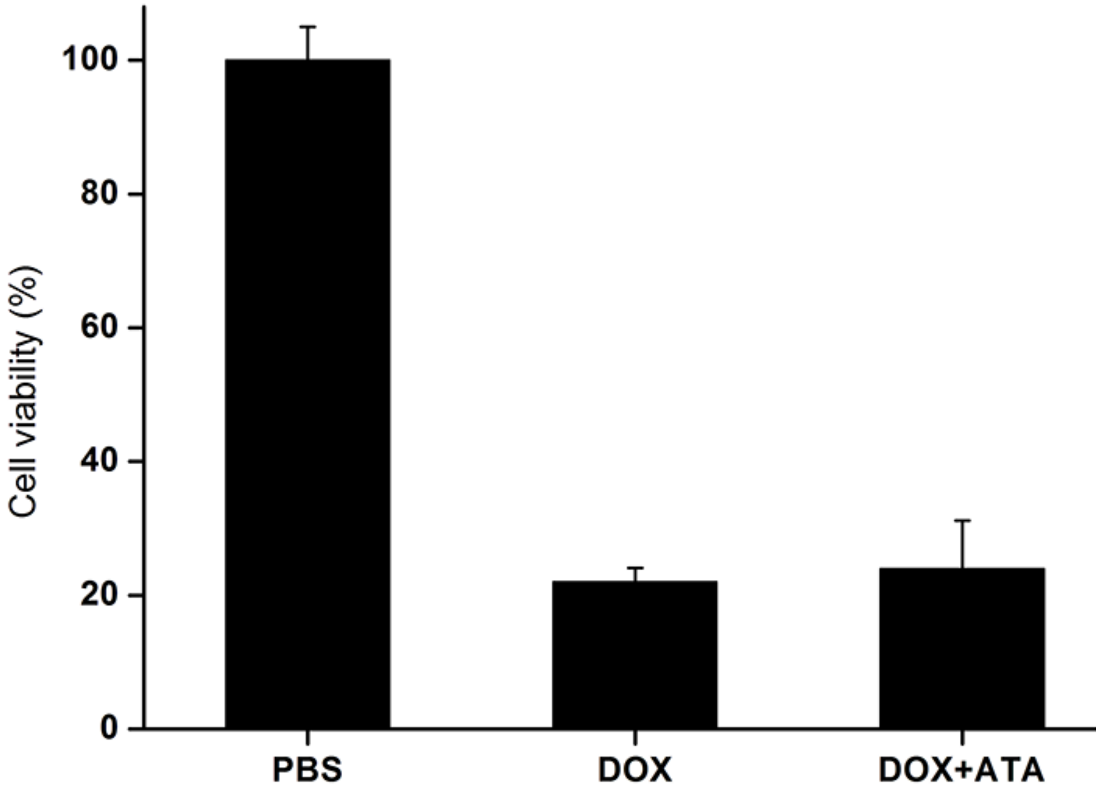

Figure S3: HepG2 cell cultures (1× 10^6 per well) were incubated with 0.2 μg/ml Dox with or without 1 μM ATA for 12 hours before the cell viability were evaluated. The experiments were triplicated and the results are expressed as mean ± SE.
